# Patient Referral Patterns and the Spread of Hospital-Acquired Infections through National Health Care Networks

**DOI:** 10.1371/journal.pcbi.1000715

**Published:** 2010-03-19

**Authors:** Tjibbe Donker, Jacco Wallinga, Hajo Grundmann

**Affiliations:** 1 Centre for Infectious Disease Control, National Institute for Public Health and the Environment, Bilthoven, The Netherlands; 2 Medical Microbiology, University Medical Center Groningen, Groningen, The Netherlands; 3 Julius Center for Health Research and Primary Care, University Medical Center Utrecht, Utrecht, The Netherlands; University of Texas at Austin, United States of America

## Abstract

Rates of hospital-acquired infections, such as methicillin-resistant Staphylococcus aureus (MRSA), are increasingly used as quality indicators for hospital hygiene. Alternatively, these rates may vary between hospitals, because hospitals differ in admission and referral of potentially colonized patients. We assessed if different referral patterns between hospitals in health care networks can influence rates of hospital-acquired infections like MRSA. We used the Dutch medical registration of 2004 to measure the connectedness between hospitals. This allowed us to reconstruct the network of hospitals in the Netherlands. We used mathematical models to assess the effect of different patient referral patterns on the potential spread of hospital-acquired infections between hospitals, and between categories of hospitals (University medical centers, top clinical hospitals and general hospitals). University hospitals have a higher number of shared patients than teaching or general hospitals, and are therefore more likely to be among the first to receive colonized patients. Moreover, as the network is directional towards university hospitals, they have a higher prevalence, even when infection control measures are equally effective in all hospitals. Patient referral patterns have a profound effect on the spread of health care-associated infections like hospital-acquired MRSA. The MRSA prevalence therefore differs between hospitals with the position of each hospital within the health care network. Any comparison of MRSA rates between hospitals, as a benchmark for hospital hygiene, should therefore take the position of a hospital within the network into account.

## Author Summary

The prevalence of hospital acquired infections is widely believed to reflect the quality of health care in individual hospitals, and is therefore often used as a benchmark. Intuitively, the idea is that infections spread more easily in hospitals with a poor quality of health care. This assumes that the rate at which admitted patients introduce new infections is the same for all hospitals. In this article, we show that this assumption is unlikely to be correct. Using national data on patient admissions, we are able to reconstruct the entire hospital network consisting of patients referred between hospitals. This network reveals that university hospitals admit more patients that recently stayed in other hospitals. Consequently, they are more likely to admit patients that still carry pathogens acquired during their previous hospital stay. Therefore, the prevalence of infections does not only reflect the quality of health care but also the connectedness to hospitals from which patients are referred. This phenomenon is missed at the single hospital level; our study is the first to address the connectedness between hospitals in explaining the prevalence of hospital acquired infections. Our findings imply that interventions should focus on hospitals that are central in the network of patient referrals.

## Introduction

Pathogens that typically cause hospital-acquired infections have an opportunistic nature. These organisms are usually part of the normal bacterial flora of humans and only cause disease when reaching body sites that are normally free from bacterial colonization e.g. when anatomical barriers are breached due to trauma or medical/surgical interventions. For this reason, severe problems with nosocomial pathogens are mainly seen in the very young and elderly and most frequently in institutions such as hospitals and long-term care facilities where patients are treated for acute or chronic conditions.

Methicillin-resistant *Staphylococcus aureus* (MRSA) is an antimicrobial resistant variant of *S. aureus*, a common bacteria frequently colonizing healthy humans and animals. Emergence of MRSA is due to the acquisition of a large DNA fragment, which seems to be rare [1],[2]. The expansion of a limited number of MRSA clones that characterizes the current epidemic in hospitals worldwide is therefore believed to be the result of between patient transmission and only to a minor extent due to the ‘de novo’ emergence in patients exposed to antibiotics. MRSA has therefore become the marker with which the success or failure of hospital infection control [3].

The prevalence of the MRSA differs considerably within and between countries [4],[5]. Currently about 30% of the *S. aureus* causing bloodstream infections in the UK is resistant to methicillin, against only 1% in the Netherlands and Scandinavian countries [6]. Although in high endemic countries MRSA infections are frequent in all hospitals, the proportions are highest in large teaching (tertiary care) hospitals [4],[7], which also report the highest frequency of newly occurring MRSA clones [8]–[11]. The severity of underlying medical condition of the patients, as well as higher antibiotic use and frequency of invasive procedures have been proposed as the main reasons for this difference [3].

Patients can carry MRSA, asymptomatically, for a long time [12]. When readmitted, they may introduce the pathogen acquired during a previous admission into a new hospital [13]. Failure of one hospital's infection control measures can therefore affect the prevalence in hospitals with which it shares patients [14]. Patients are referred to hospitals at different rates depending on the function of hospitals within the health-care system, which likely affect the prevalence at different institutions. These referral patterns might therefore offer an explanation for high MRSA incidence in hospitals of the tertiary referral level [7]. But can referral patterns account for differences in spread between hospitals, and for differences in observed prevalence? To answer these questions, we have been mapping the health care network based on a large national medical registry, and evaluated the occurrence of hospital-acquired infections in different care categories under simulated epidemic conditions.

## Results

**Figure 1:**
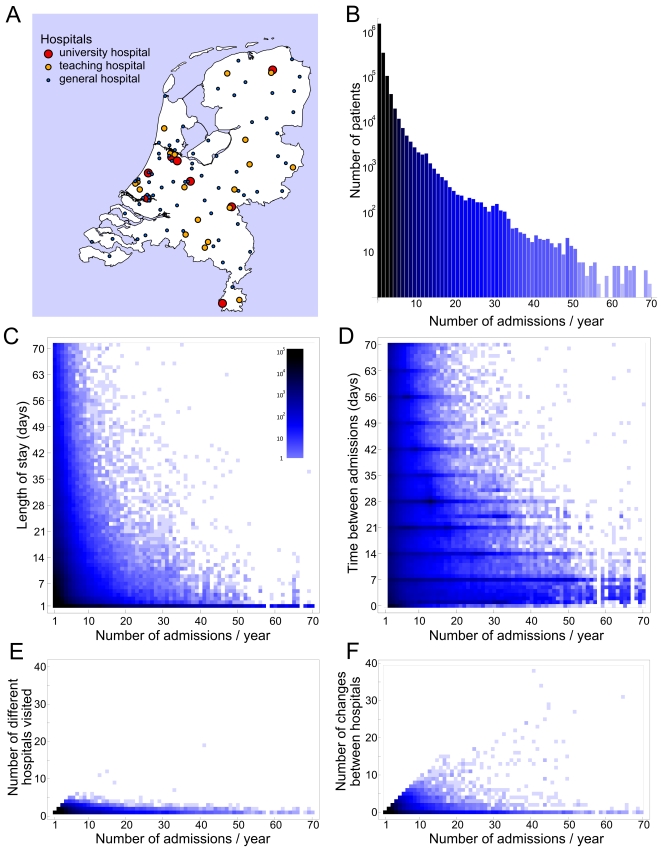
Health care utilization in the Netherlands in 2004. A) Map of the Netherlands showing the location of the university medical centers, top clinical and general hospitals. Patients were stratified based on the number of admissions in one year, and per stratum we measured: B) the number of patients and the distributions of C) the length of stay, D) time between admissions, E) number of different hospitals visited and F) the number of changes between hospitals, *i.e.*, the number of admissions in a different hospital than the previous one.

In 2004, hospital care in the Netherlands was provided through 71 general hospitals, 19 top clinical hospitals and 8 university medical centres (Figure 1A). During the observation period of one year (2004) 1,676,704 patients were admitted from the population of 16.7 million. These patients were admitted for a total of 2,611,452 times, the majority of patients were hospitalised once. The frequency with which patients were readmitted showed a right-skewed distribution (Figure 1B), with still 86 patients being readmitted for more than 52 times. Patients stayed on average 4.3 days per hospital admission, patients who had less hospital admissions stayed longer per admission (Figure 1C), and those who had four hospital admissions had on average the longest (5.6 days) episodes of hospital admission. Moreover, these patients had the highest rate of readmission in different hospitals (Figure 1E&F), whereas patients who were readmitted more frequently tended to return to the same hospital. These frequent attendees were also most likely to stay for only one day.

**Figure 2:**
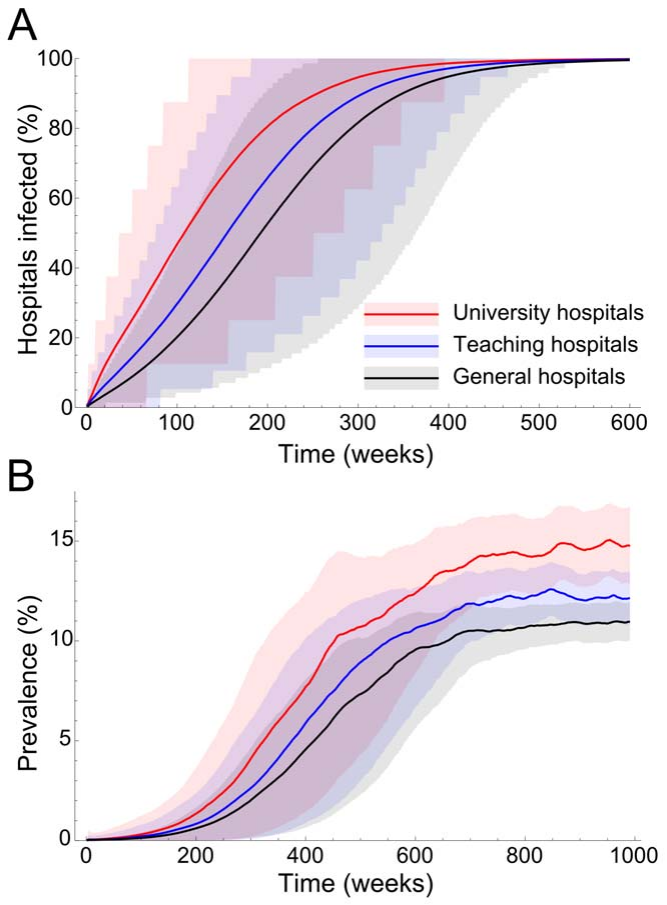
Spread of hospital-acquired infection between hospitals in absence of interventions, according to our individual-based model results using the recorded health care utilization patterns. The thick lines show the mean and shaded areas show all runs between the 5^th^ and 95^th^ percentile. A) Time to encounter of the first colonized patient. B) Prevalence of colonization among admitted patients.

The individual-based model emulated the dynamics of patient referrals and allows us to assess the spread of hospital-acquired infections. Colonized patients from one hospital spread the pathogen to nearby hospitals within days, but it takes more time –5 to 10 years– before all hospitals encounter it (Figure 2A). The median time to first infection (TFI) for university medical centers (UMCs) was 755 days, the TFI for top clinical hospitals was 1,087 days and the TFI for general hospitals was 1,346 days. At any stage of the epidemic the expected prevalence in UMCs was higher than in general and top clinical hospitals (Figure 2C).

**Figure 3:**
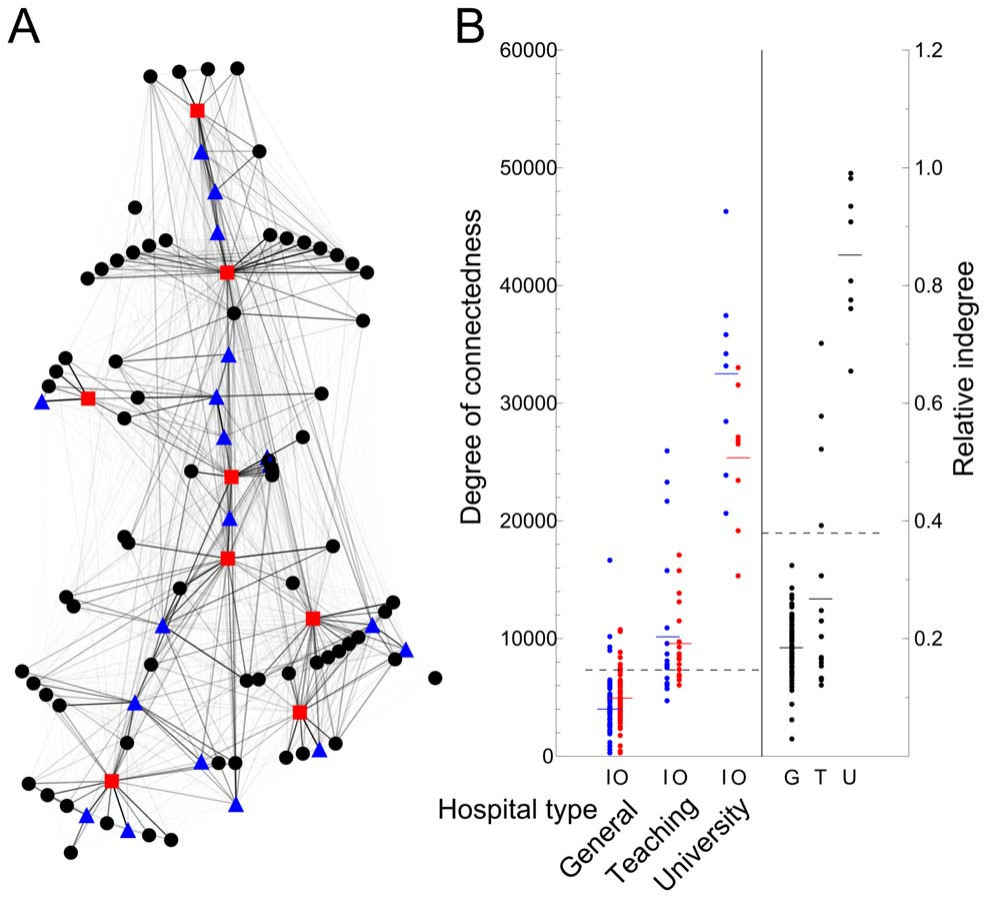
Patients referred between hospitals in the Netherlands. A) The reconstructed Dutch health care network based on the potential infection rate between hospitals, red squares denote university medical centers, blue triangles the top clinical and black circles the general hospitals. B) Inward (blue), outward (red) and relative inward (black) degree of connectedness per hospitals category, calculated from the Dutch medical registration. The relative indegree is the indegree divided by the total number of admissions. Solid lines show mean degree per category and the dashed line shows the overall mean degree. University medical centers take a clear central position, in the sense that they have a high degree of connectedness. The network is directional towards the UMCs as they have a higher indegree than outdegree.

We reconstructed the Dutch national network of hospitals (Figure 3A) with respect to the potential spread of hospital-acquired infections, using patient referral patterns taken from national medical registration (LMR [15]). Within this network, the UMCs show a higher degree of connectedness than the general and top clinical hospitals (Figure 3B). General hospitals had a higher outdegree than indegree, whereas the reverse was true for UMCs, resulting in an 8-fold difference in the indegree between both types of institutions. Top clinical hospitals assumed an intermediate position and showed little difference between indegree and outdegree. Moreover, the indegree relative to the total number of admissions (including patients admitted directly from the community) was much higher in the UMCs compared to the general hospitals. The patient flow through the network was thus directed towards the UMCs.

**Figure 4:**
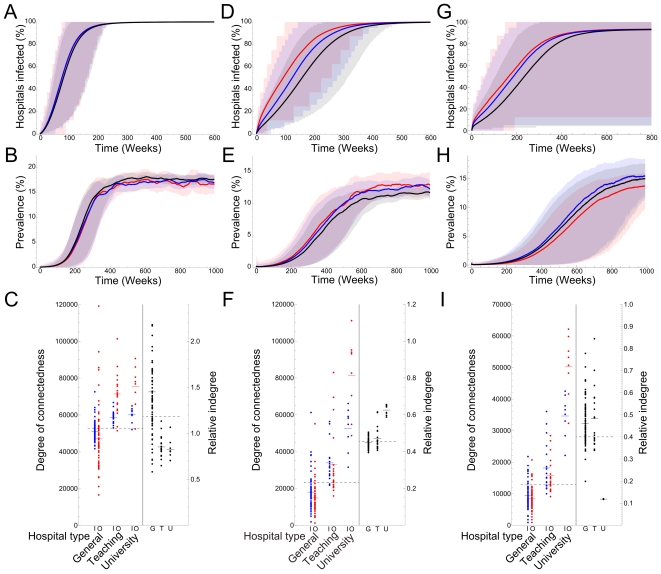
Impact of directionality of the hospital network on the spread of hospital acquired infections. We created three networks with alternative directions. One with equal referral probabilities to all other hospitals (A, B & C), one exact reversion of the original network (D, E & F) and one with an exaggerated reversion of the direction (G, H & I). A, D & G show the time to encounter of the first colonized patient, B, E & H show the prevalence among admitted patients and C, F & I show the indegree (blue), outdegree (red) and relative indegree (black). Reversion of the network direction leads to a lower prevalence in university medical centers, while they are still the first to encounter the infection, showing that the relative indegree, the indegree divided by the total number of admissions, relates to the found prevalence and the high absolute indegree relates to the time to first encounter.

In order to determine the effect of the directionality of the network, we repeated the analysis of the individual-based model using a dataset with alternative direction. We created a dataset in which all referral probabilities to hospitals were set equal. In the resulting network, both the indegree and outdegree of the UMCs were higher than the other hospital categories, but the outdegree is now higher than the indegree (Figure 4A, B & C). The relative indegree was higher for the general hospitals compared to the other two categories, although there was only a small difference between UMCs and top clinical hospitals. These simulations resulted in slightly higher prevalence in the general hospitals, compared to the top clinical hospitals and UMCs. The differences between the hospitals in connectedness and prevalence are caused by the different hospital sizes, the only parameter that varied between hospitals in this model. This suggests that the short time to first infection of UMCs is due to their absolute high degree of connectedness, while their high relative indegree causes the higher prevalence in UMCs relative to other hospital categories.

We also used two other networks with alternative directions, to test if our observation holds under different conditions. First, we reversed the direction of the network by reversing time in the original dataset, the patients who first visited a general hospital and then a UMC now do the opposite. In this dataset the UMCs still have a higher relative indegree, compared to the general hospitals, although their outdegree is now higher than their indegree (Figure 4D, E & F). These simulations reduced the difference in prevalence between hospitals, with still the highest prevalence in the UMCs. This exact reversion had almost no effect on the TFI of all hospital categories.

Second, we increased the reversed direction in order to decrease the relative indegree of the UMCs to a level below the relative indegree of the general hospital, while keeping both the absolute degree of the UMCs (both indegree and outdegree) above the degree of the general hospitals. These simulations resulted in a lower prevalence in the university medical centres compared to the hospitals of other care categories, whereby the top clinical hospitals had the highest prevalence, reflecting their highest relative indegree (Figure 4G, H & I). This reversion of direction in the network had, just like the previous ones, little effect on the order of TFI for the hospital categories. The results of all three simulation studies with alternative directions, when taken together, strongly suggest that the high prevalence in UMCs relative to other hospital categories is due to directionality of referral patterns, reflected by their high relative indegree.

## Discussion

This study sets a precedent by using data about all hospital admissions obtained from the National Medical Register (LMR [15]) to explore the potential spread of hospital-acquired infections through the Dutch national network of hospitals and describing the effect of nationwide referral patterns on the spread of nosocomial infections like MRSA. This method shows properties of hospitals, such as connectedness within the network, that on the level of a single hospital would not be visible.

In the Netherlands, 98 hospitals provide various forms of specialist care. Within the category of general hospitals, there are considerable differences from hospital to hospital, with some smaller hospitals providing only basic hospital care. Therefore, patients who need advanced medical treatment need to be referred to so-called top clinical hospitals or university medical centres. Top clinical hospitals are large institutions that provide a wide range of clinical specialities and are involved in specialists training and education of doctors and other health care workers. In contrast to university medial centres they are not affiliated with universities and do not include the same comprehensive spectrum of specialities. Within the health care system, the university medical centres occupy a special place as leading hospitals with advanced specialist and final referral functions.

In the Netherlands the hospital admission rate is rather low compared to international standards with 15.6 admissions per 100 inhabitants [16] and an average stay of only 4.3 days. This figure is low, as it also includes day care treatment when patients occupied a bed but do not stay overnight. The majority of patients (73%) are admitted only once to any hospital. Few return twice (17%), three times (5%), or more (5%). Importantly, patients who are admitted twice or three times in a one year period not only have the longest per admission treatment episodes, but are also more frequently readmitted to different hospitals. In this way, all hospitals in the Netherlands become connected and form a network consisting of referred patients who form a bridge between hospitals and provide a path that can facilitate the spread of hospital-acquired infections, such as MRSA, between hospitals.

The individual-based model which emulates the referral characteristics recorded in the LMR, describes the spread of nosocomial infections among hospitals on an individual patient level. It shows that patients who are admitted only two or three times contribute significantly to the inter-hospital spread of the infection and suggests that the prevalence is directly related to the referral level of different hospital categories. This model is, however, unable to provide a mechanistical explanation for the predicted differences in prevalence between hospital categories. For this reason, a simplified model of the hospital network was created. This model weights the contact pattern between hospitals on the basis of average patient referrals between any two hospitals without taking individual referrals and catchment populations into consideration. Despite being a coarse simplification, the hospital network model provides excellent heuristic value as it is able to demonstrate the directionality of the entire network, which is the driving force behind the difference in prevalence between different hospital categories.

Our methods rely on three key assumptions that should be addressed. First, all of our methods do not take account of transmission outside of the hospitals. If community transmission of hospital-acquired infections become a significant factor, the dynamics of the epidemic will ultimately change and the effect of patient referrals between hospitals will be diluted. Community transmission of MRSA is mainly seen in families [17], among military recruits [18], in relation with competitive sport activities [19] and among children in day-care centres [20]. Typical community-acquired (CA-) MRSA is a phenomenon widely described in the USA [21]–[24] but still rather uncommon in Europe. Although CA-MRSA has been identified in Europe in countries with high as well as low MRSA prevalence, it so far remains much less prevalent than health-care associated (HA-) MRSA. Indeed a recent comprehensive study among patients consulting general practitioners in the Netherlands could not find any CA-MRSA in this population [25]. For MRSA, our models will lose validity when CA-MRSA becomes widespread in the general population and the prevalence in the population reaches levels comparable with those in hospitals.

Second, we have assumed a specific measure of connectedness to create the network. However, the construction of hospital networks can be done based on other measures than the one we used, like weighting the contact between two hospitals by the number of patients these hospitals share, or by taking only subsequent admissions into account. These measures would slightly alter the difference in connectedness between the hospital types, but the differences between referral levels would remain (data not shown). However, we feel that exclusion of data about the length of stay and time between admissions would disguise the true utilization patterns that govern the spread of HA-MRSA.

Third, both the individual based model and the measure of connectedness assume homogeneous mixing within the hospital and leave out any ward structure. However, because the medical condition of a patient determines both the ward of admission and his/her health-care use, patients with a certain utilization pattern may mainly meet patients with comparable utilization patterns. This assortative behavior of patients [26] can potentially alter the dynamics of the epidemic, and especially the rate of growth of the epidemic. However, although the different wards may show different dynamics with the different patients they admit, the general direction of the referred patients will still be towards the university hospitals. We therefore expect the difference between hospital categories to still hold in the long run, despite some likely transient effects during the growth of the epidemic.

A higher prevalence of health care-associated infections has been repeatedly demonstrated for tertiary referral centres such as university and teaching hospitals, which also witness the majority of outbreaks of these types of infections. As a conventional explanation, the severity of underlying conditions, more invasive diagnostic and therapeutic procedures and higher rates of antibiotic prescription have been incriminated for this difference. Our model predictions based on the observed admission pattern in the Netherlands, however, suggest a more parsimonious explanation. In the Dutch health care network, the university medical centres admit a large number of referred patients from other hospitals, much more than the top clinical hospitals (Figure 3B). Each university medical centres is therefore connected to a large number of general hospitals as well as a number of top clinical hospitals. This central position within the hospital network puts these hospitals at higher risk of encountering colonized patients. Moreover, the flow of infectious patients through the hospital network is directed towards the university medical centres and we could show that as a direct result of this directionality, prevalence in these hospitals is predictably higher relative to the other categories.

These observations can have important implications concerning hospital infection control. When hospital infection control fails within a single hospital, hospital-acquired infections will start to spread between hospitals, with the most connected ones at the highest risk of both acquiring and spreading the disease. Differentiation of intervention measures over hospital categories, for instance by making the university medical centres the focal point, could then be considered. The exact implementation of such a differentiation is, however, beyond the scope of this paper and should be the focus of further research. Furthermore, our results suggest that differences in prevalence of nosocomial infections between hospitals do not necessarily reflect the success of the hospital infection control measures of individual hospitals. Direct comparisons of infection rates between hospitals may therefore give a distorted view of hospital standards, if national (or regional) health care utilization patterns are not considered. The use of such comparisons, for benchmarking, may therefore lead to a false conclusion about a hospitals effort to reduce nosocomial infections.

In summary we predict that (1) Hospital-acquired infections can spread rapidly from index hospitals to the next referral level. (2) Secondary and tertiary referral hospitals must be prepared for rapid response. (3) High connectedness and the directionality in the health care network towards the university medical centres cause a local build-up of nosocomial pathogens, such as MRSA, and thus a higher prevalence in these hospitals. This should be taken into consideration for benchmarking and the design of national control strategies.

## Materials and Methods

### Generating a simulated dataset

We used the Dutch national medical register from 2004 (Landelijke Medische Registratie LMR [15]), which contains the data about all individual hospital admissions for the total of Dutch hospital organizations of that year. We stratified patients in the LMR based on the number of admissions, 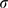, in the one year of data. Per stratum we counted the number of patients, 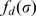, and measured the distribution of the length of stay, 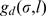, the time between admissions, 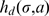, number of hospitals visited, 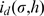, and the changes between hospitals, 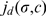. We defined a change between hospitals as an admission to a hospital different from the hospital of the previous admission. For each hospital 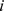 we counted the number of next admissions in other hospitals 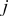 to determine the referral probability, 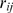, and counted the the number of admissions per hospital to determined the size, 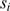.

For reasons of privacy protection, we were not authorized to use the data at individual record level for detailed analysis. We therefore generated a simulated dataset based on the recorded frequencies which describes the individual patient referral patterns that is consistent with the observed patient characteristics in the LMR. This also enabled us to expand the simulated dataset beyond the recorded single year in the LMR to 20 years.

We assumed that each patient's health-care use comes in sequences of a given number of hospital admissions, 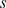, and that the time between these sequences, i.e. between the moment of discharge of the last admission in the sequence and first admission in the next sequence, is exponentially distributed. Patients were assigned a hospital of initial admission from the hospital size distribution, 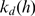, and a number of admissions in this sequence 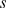 from distribution 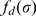. The number of changes between hospitals during these admissions was picked from the distribution 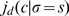. If the number of changes was larger than 0, the same was done for the number hospitals visited, picked from the distribution 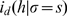. We assumed that the moment of changing between hospitals was distributed uniformly over the admissions and the choice for the new hospital was based on the current hospital's referral distributions. The length of stay was picked from distribution 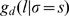 and time between admissions from distribution 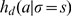 for all sequential admissions.

We picked the rate of initial admission, 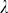, based on over 1.6 million admitted patients for an entire population of 16 million individuals, at 1/3650 day^−1^. After the last admission in the sequence, the time to next admission is therefore picked from an exponential distribution with mean 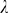. Because the average time between admission sequences is much longer than the average length of colonization, we thus assumed that the colonization status of an individual at the start of an admissions sequence does not depend on this individuals colonization status in the previous admission sequence. We created a dataset for 20 years to allow the epidemic to reach equilibrium level.

### Individual based model

Using the individual entries of the simulated dataset we subsequently created a mathematical model that describes the effect of individual patient movements through the hospital network on the spread of hospital-acquired infections. These individuals can either be susceptible or infected. No distinction was made between colonization and clinical infection for the sake of simplicity. Infected individuals (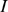) infect susceptible individuals (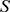) within the same hospital during one day with rate 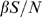, where 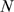 is the total number of patients in the hospital. Therefore, each susceptible has a probability of 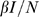 of getting infected per day. We assume that infectious patients spread the infection to a random sample of the patients within the hospital, and take no ward structure into account. Individuals lose the infection with rate 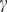 and the mean duration of colonization 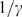 was set at 365 days [12].

In order to explore the dynamics, we infect 10% of the patients that are admitted to an index hospital on a randomly chosen starting date, and monitor how the infection spreads to other hospitals. The number of colonized individuals at each time step and the time to first encounter of a colonized patient in each hospital (time to first infection, TFI) was recorded. For each index hospital we perform 200 simulations, sequentially repeating these sets of simulations for each 98 hospitals as index hospital, thus performing a total of 19600 simulations. In further analysis, we only include simulation runs resulting in an outbreak larger than a threshold of 1000 colonized persons, to exclude runs that resulted only in small local outbreaks. The results are not sensitive to the exact value of this threshold.

### Contact matrix

In order to reduce the complexity inherent to the individual-based model, we created a hospital network model assuming transmission parameters between hospitals. All transmission parameters were based on the patient characteristics as observed in the LMR. Thereby, we calculated the infection rate, 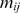, from hospital 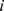 to hospital 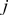, using the probability that any referred patient transmits the infection after referral. This probability depends on the patient's length of stay in both hospitals and the rate of losing colonisation between admissions. The infection rates between all hospitals form a 98
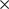
98 matrix, 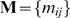, which describes the national network of hospitals in terms of potential transmission.

For each admission we calculate the probability that the patient transmits the infection from the referring hospital to the admitting one, 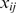. This probability can basically be divided into three separate probabilities: contracting the infection in a referring hospital, 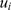, still being colonized on readmission, 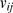, and spreading the infection in the admitting hospital, 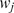:
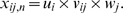
(1)


The probability of being colonized depends on the length of stay in each referring hospital, 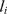, the number of colonized patients in each of these hospital, 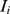, and the transmissibility of the pathogen, 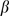; 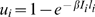. If we assume that both the infectivity and the number of colonized patients are at a fixed low level, we can simplify this to 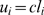, where 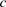 encompasses the transmissibility and low prevalence in the hospital. Because we assume the transmissibility and prevalence are equal in all hospitals, and because the matrix scales linearly with 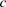 we can leave 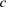 at unity:
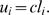
(2)


The probability of introduction in the admitting hospital, 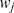, in turn depends on the length of stay in the admitting hospital, 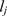, the number of susceptible patients, 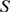, and the transmissibility of the pathogen, 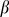; 
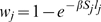
. Here, we can assume that the number of newly infected patients is not dependent on the size of the hospital, because ward size is generally not related to hospital size. Therefore, the probability of transmission is directly related to the basic reproduction number per admission, 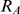, and becomes 
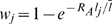
. Where 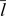 denotes the average length of stay in the dataset. Just as before, we assume that the number of colonized patients is low, and the process is not limited by the number of available susceptible individuals:
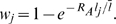
(3)


The probability that a patient is still colonized upon readmission, 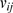, depends on the time between discharge and admission, 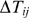, and the recovery rate, 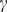; 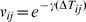. Although overlapping admissions do occur in the data –patients can for instance be moved to another hospital for a specific procedure without being discharged from the initial hospital– we simplify by only taking sequential admissions into account. Any overlapping admission is treated as having a time between admissions, 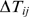, of 0, thus with 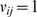:
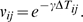
(4)

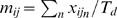
 gives the infectious referral rate, per day, between hospitals, where 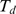 denotes the time span of the dataset. 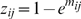 now denotes the probability that any patient will transmit the disease from hospital 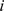 to 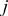 within one day. All admissions of all patients combined result in the national hospital network 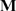.
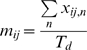
(5)

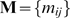
(6)


The degree with which hospitals connect with the rest of the hospital network through referrals of patients can be divided into two parts. These consist of the indegree 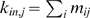, reflecting the total of introductions a single hospital receives from the rest of the hospital network, and the outdegree 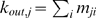 which reflects the total amount of colonized patients a single hospital exports to the rest of the hospital network. Because the matrix 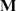 is asymmetric, 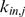 and 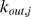 may differ.

### Alternative direction datasets

In order to determine the effect of the difference between inward and outward degree of connectedness, we created a number of datasets with alternative directions. One of these has no direction, the other two have reversed directions. In all three alternatives the university medical centers still have a high degree of connectedness, consistent with the LMR-based network, but a higher outdegree than indegree, contrary to the LMR based network.

We first created a dataset without direction, by setting all referral probabilities in the referral matrix equal, but leaving all other parameters the same as the original simulated dataset. We then created a reverse dataset by reversing the time of the original simulated dataset. The new date of admission of a patient, 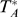, is simply calculated as 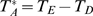, where 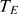 is the end date of the dataset, in our case day 7300, and 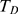 is the discharge date of the patient. This then gives the exact reversion of the original simulated dataset.

In order to reverse the direction of the dataset even further, we created another dataset in the same way as the generated dataset with the characteristics of the LMR, in which we set all referral probabilities to university medical centers, in the referral matrix, to zero. This, however, also lowered the overall degree of connectedness of these hospitals. In order to raise the degree we increased the size of the university medical centers 7 fold. The university medical centers now have a higher outdegree than indegree, while their indegree is still higher than the outdegree of the top clinical hospitals.

Furthermore, we created a number of small datasets of only five hospitals, in which we varied network properties such as directionality and hospital size (See Text S1).

## Supporting Information

Text S1 Analysis of networks consisting of five hospitals in which network properties, such as directionality and hospital size, are varied.
(7.71 MB PDF)
