# Supplementary material for: Patient Referral Patterns and the Spread of Hospital-Acquired Infections through National Health Care Networks
Source: PLoS Comput Biol. 2010 Mar 19;6(3):e1000715. doi: 10.1371/journal.pcbi.1000715 (PMC2841613; doi:10.1371/journal.pcbi.1000715)
Supplement: Text S1 — Analysis of networks consisting of five hospitals in which network properties, such as directionality and hospital size, are varied. (7.71 MB PDF) [file pcbi.1000715.s001.pdf]

Text S1

To further explore the effects of the parameter settings in the models and to assess the influence of the network directionality on the spread of Hospital Acquired Infections (HAIs), we created a smaller network of 5 hospitals (Figure S1A). We used this network to generate a number of datasets based on different parameter settings, but always using the measured patient health care utilization. The datasets are therefore created in the same way as the simulated dataset of the Dutch network, apart from the parameters for referral probability and hospital size. Moreover, because we have less hospitals (5 in stead of 98) we also reduced the total population size from 16 million to 0.8 million.

For each of the simulated datasets we determined the desired relative hospital sizes and referral probabilities (Figure S1B), and then created it in the same way as the Dutch simulated dataset. From the simulated dataset we measured the contact matrix (Figure S1C), which describes the network and is used for the calculation of the indegree and outdegree (Figure S1D). Furthermore, we run the individual-based model on the simulated dataset 100 times for each of the five index hospitals.

During each simulation, we measured the prevalence in each hospital (Figure S1E) and excluded any simulations with less than 50 colonized individuals at the end of the time-frame. However, because we measure the prevalence of each hospital individually and have a relatively small population size, even the mean over all runs will show some stochasticity (Figure S1F) due to the patient movements in the dataset. We therefore decided to show the difference in the distribution of prevalences during the equilibrium phase (Figure S1G). We chose 500 weeks (i.e., 9.6 years after the introduction) as the start of the equilibrium phase to include as much data as possible, but still exclude as much of the growth phase as possible. Furthermore, we performed the procedure five times to show the influence of a single simulated dataset.

We display the referral matrix as a “relative referral matrix”, which is not yet normalized to referral probabilities. where the entries are scaled such that the smallest off-diagonal entry is 1. We display the hospital size distribution, as relative size, where the smallest size is 1.

Directionality

To test the influence of the directionality on the spread of HAIs, we changed the referral probabilities while keeping the hospital sizes equal. We set the referral probability to the first hospital from all other hospitals larger than between all other hospitals. Furthermore, we vary the strength of the directionality by using a 2, 5, 10, 20 and 50-fold difference in referral probability between the first and other hospitals (See figure

S2).

As the directionality increases, the difference in equilibrium prevalence between the first hospital (mimicking a University Medical Center (UMC)) and the other (general) hospital becomes larger. It should be noted that the increase in difference is not only caused by an increase in equilibrium prevalence in the university hospital, but also by a decrease in prevalence in the other hospitals.

Hospital Size

We also tested the influence of hospital size on prevalence differences, because UMCs are generally larger than other hospitals. In the same way as before we created five datasets, this time with equal referral probabilities between all hospitals, but with one larger hospital. We used an equal size and a 2, 3, 4 and 5 fold difference in size between the hospitals.

An increase in size causes a narrower distribution of the equilibrium prevalence. As the hospital size increases, the individual admission and discharge events of colonized patients are having a decreasing effect on the prevalence of the entire hospital, thus reducing the stochasticity in the equilibrium prevalence (See figure S3).

Apart from that, the prevalence of the larger hospital is slightly reduced with an increasing size, while the prevalence in the other hospitals slightly increases. This effect can be explained by the number of patients referred between the hospitals and therefore the relative indegree of the hospitals. Because the larger hospital discharges more patients, that need to be distributed over the other hospitals, the relative indegree of the other four hospitals increases. Effectively it thus results in a network with reversed direction, where one larger hospital refers many patients to a number of smaller hospitals.

Increasing transmissibility, β, in the individual-based model

We also ran the model with a number of different values for β, to test the effect of differences in transmissibility of the pathogen on our model results. We used the exact five datasets created before (See Directionality section) with the largest network directionality. we increased β from 0.125 to 0.225 in steps of 0.025. As expected, an increase in prevalence can be seen with the increase of β (See figure S4). The difference between the "UMC" and other hospitals stays clearly visible, although it gets slightly smaller at higher values of β.

Reversion of referral matrix

Next we tested if the direction of the network can be reversed by transposing the referral matrix. We transposed the matrices previously used to test the influence of the strength of directionality of the network and again created five datasets per referral matrix. All of the runs showed no difference in degree of connectedness and prevalence (See figure S5).

This effect can simply be explained. A fixed part of the returning patients is admitted to a different hospital, j, than the one the were discharged from, a. The probability of being admitted to hospital j is then given by the (normalized) referral vector $r_d(j|i = a)$. With the transposing of the referral matrices, all elements in each of the vectors got the same values, thus resulting in an equal referral probability to all hospitals.

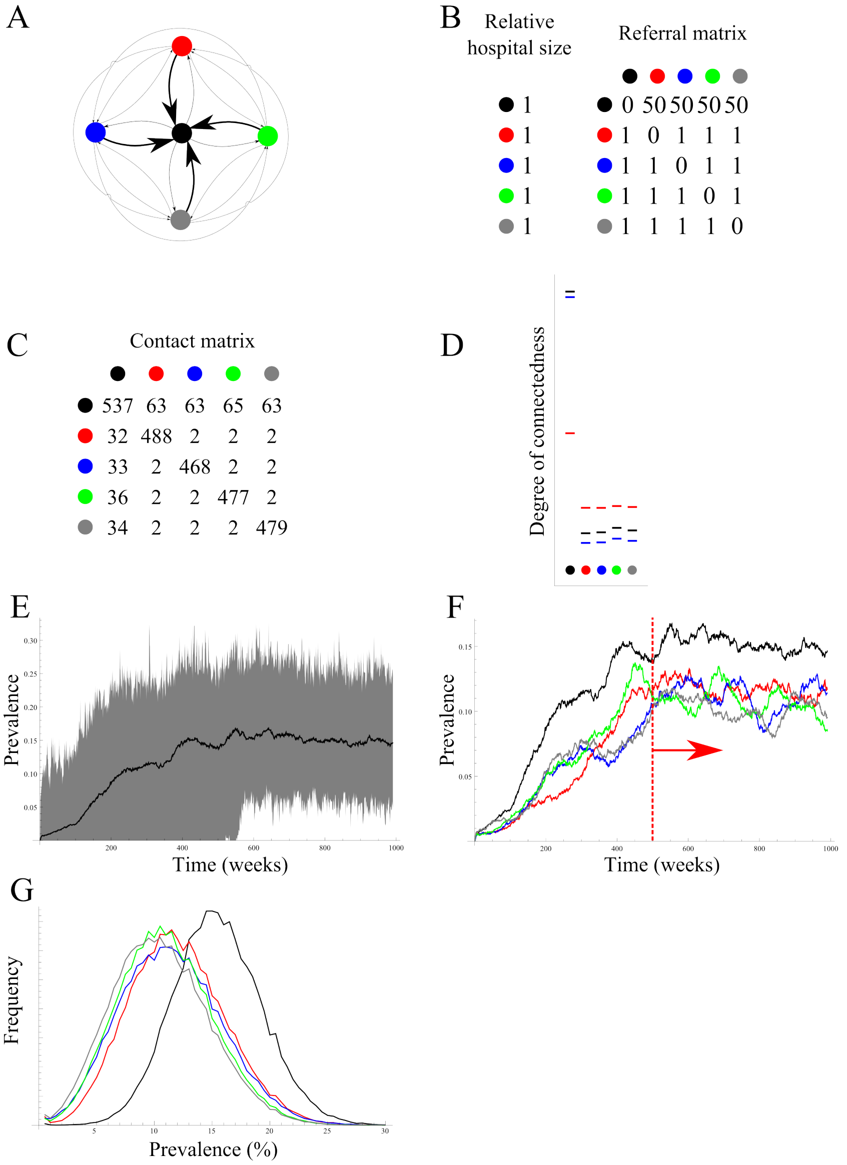

Figure S1: The construction and analysis of the networks. A) Schematic representation of a patient referral network with only 5 hospitals, dots represent the 5 hospitals in black, red, blue, green and gray. Referral directions are depicted by the arrows and the thickness of the arrows represents the referral probability. In this example the referral probability to the black hospital is higher than to all others. B) The parameters needed to simulate the dataset of the network, hospital sizes are set equal, but referral probability from red, green, blue and gray to black is, in this case, set 50 times higher than the referral probability between red, green, blue and gray. C) The contact matrix of the resulting network, showing the infectious contact rate. D) Indegree (blue), outdegree (red) and relative indegree (black) for all five hospitals. The mean relative indegree of the five hospitals is scaled to one third of the Y-axis, because its value is much lower than the absolute indegree and outdegree. E) The results from the individual based model, showing the prevalence in the black hospital, the grey area shows the range of all simulation, the thick black line shows the mean. F) The mean prevalence for all hospitals, resulting from the same individual-based simulations, the red dashed line shows the assumed start of the equilibrium phase. G) The distribution of weekly prevalence values during the equilibrium phase in all 100 repeats of the individual-based model.

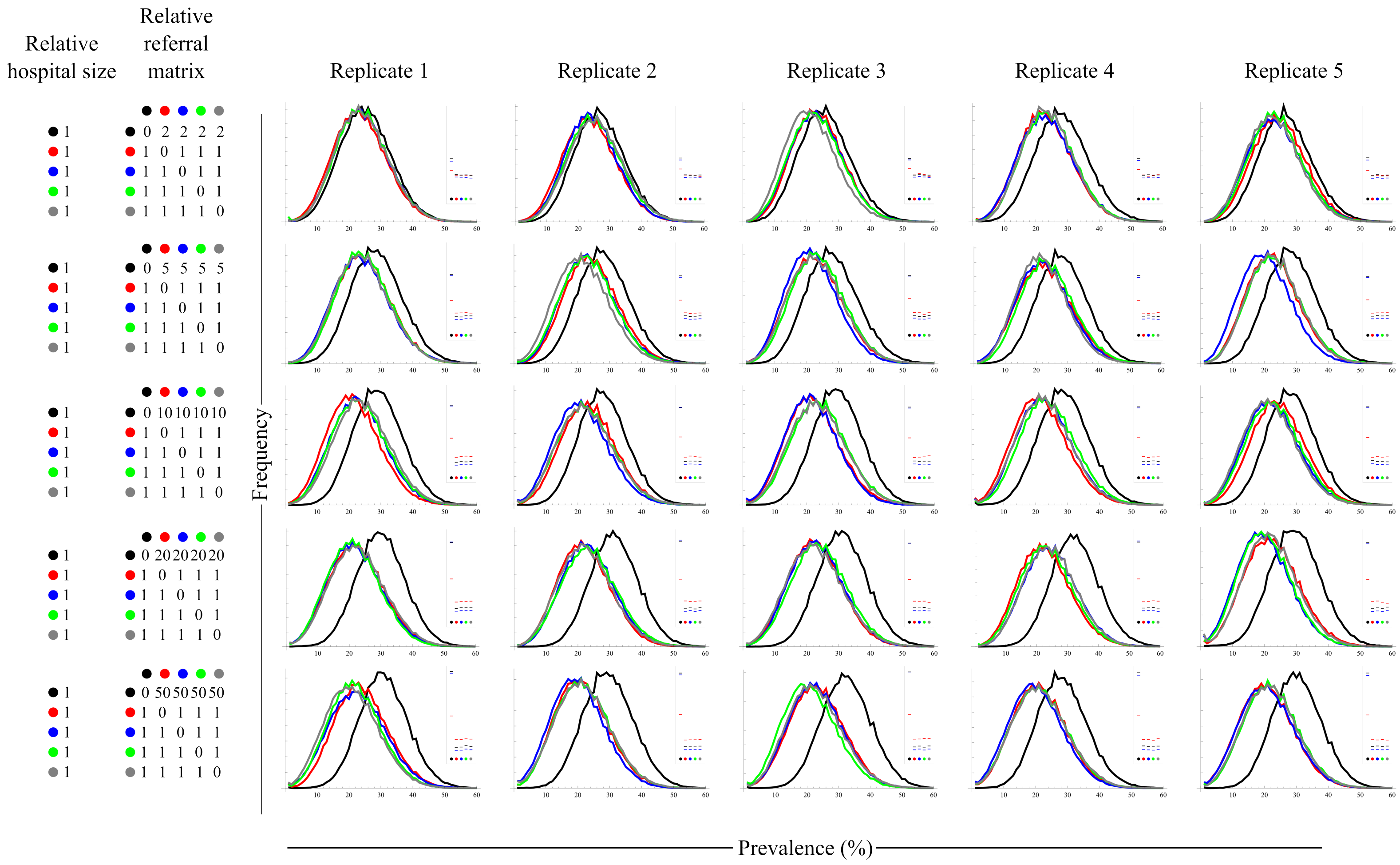

Figure S2: Effect of directionality in the patient referral network on the prevalence of hospital-acquired infections. We changed the directionality of the network by increasing the referral probability towards the black hospital, from a 2-fold (top row) to 50-fold (bottom row) difference. Hospital sizes were set equal for all hospitals. Increasing the referral probability difference increases the difference in prevalence.

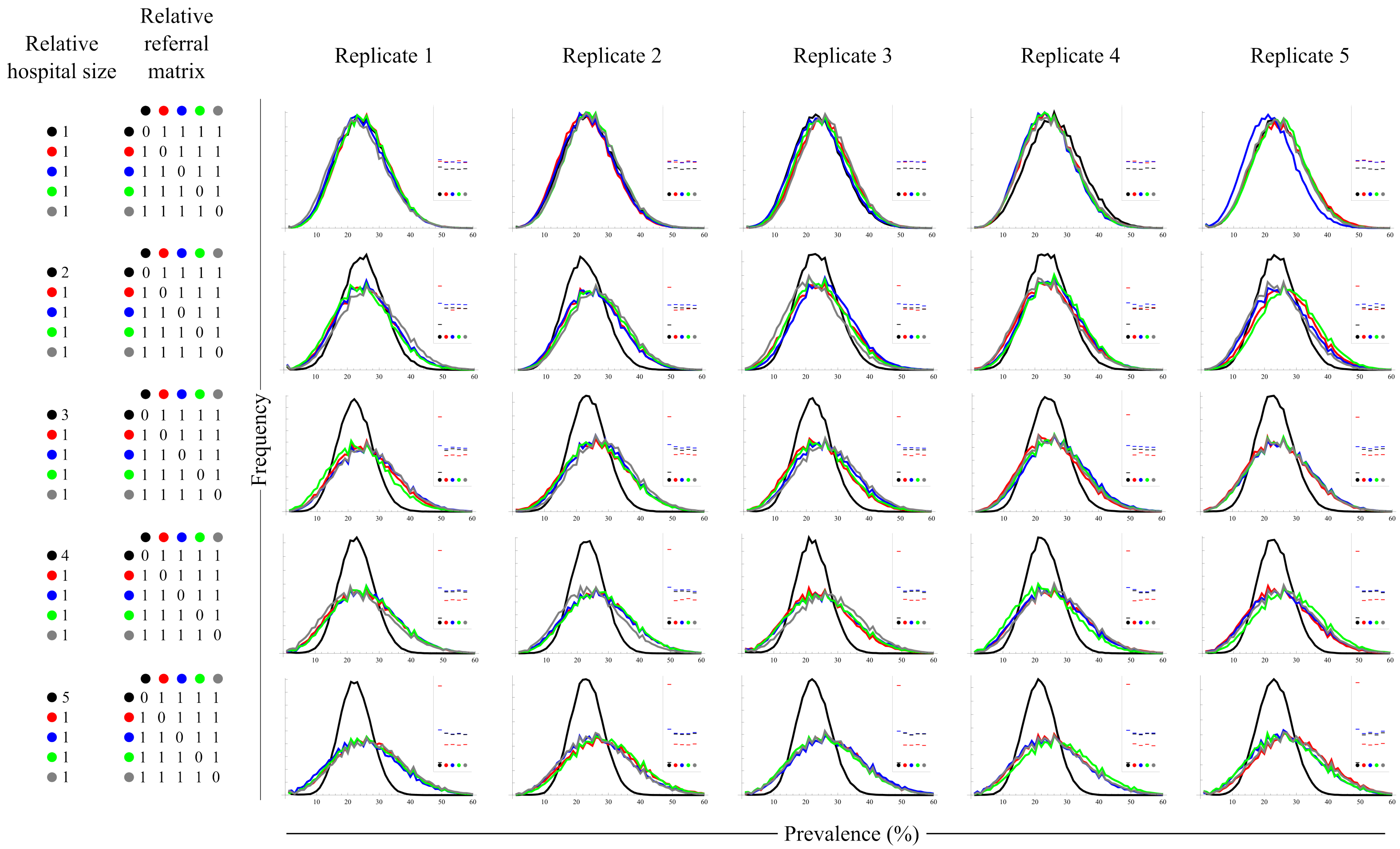

Figure S3: Effect of hospital size difference in the patient referral network on prevalence of hospital-acquired infection. We changed the size of one hospital (black) relative to the other four hospitals. We started with all hospitals at an equal size (top row) and increased the size difference to 5-fold (bottom row) The larger hospital shows less variation in equilibrium prevalence, because the individual admitted patients start to have less effect on the within-hospital prevalence. Furthermore, the prevalence of the larger hospital is lower, consistent with a lower relative indegree.

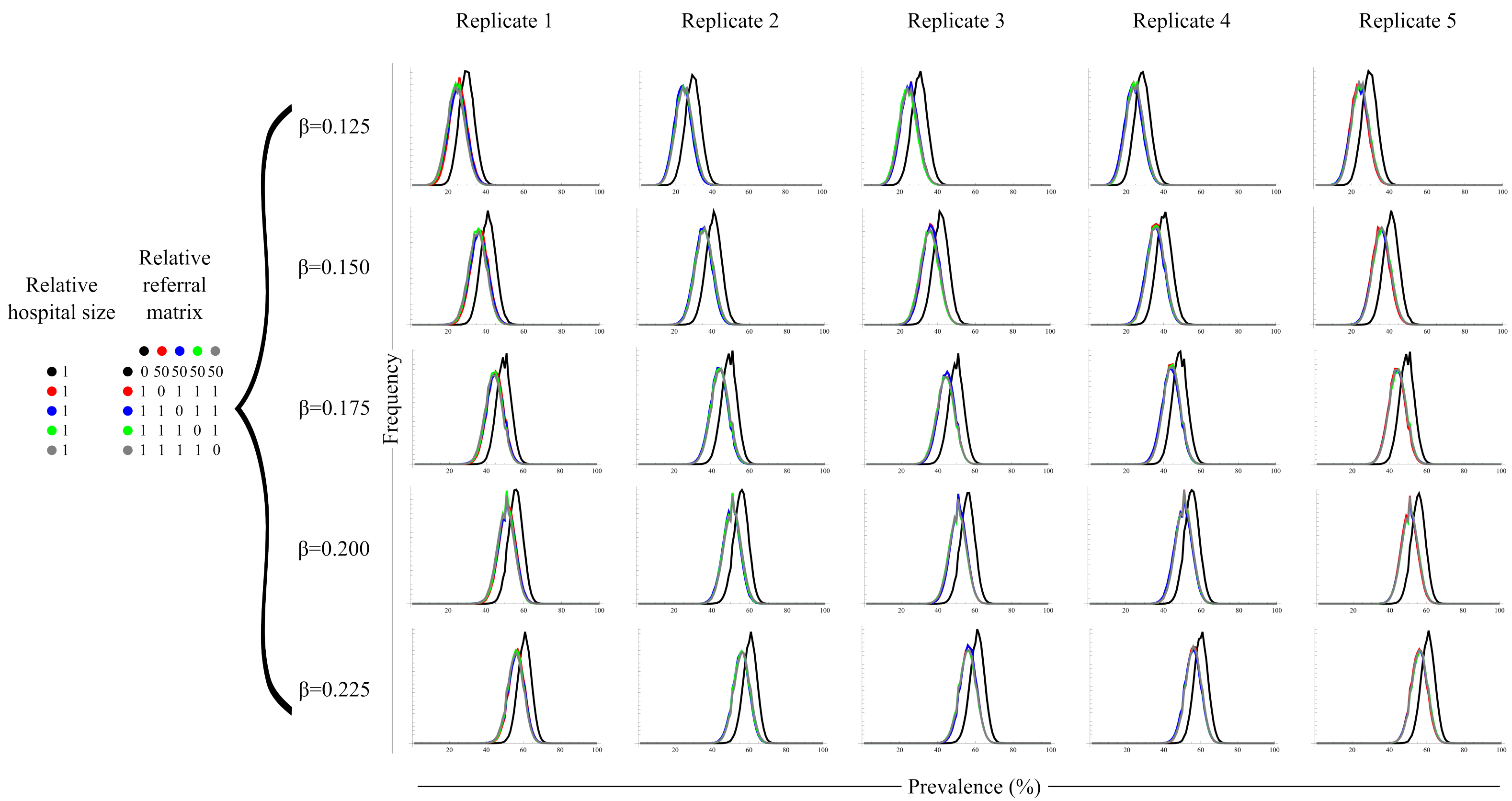

Figure S4: Effect of transmissibility on the difference in prevalence between hospitals in the patient referral network with direction towards one hospital. Using the datasets with a 50 fold difference in referral probability between one hospital (black) and the other four, we increased β from 0.125 (top row), just above our originally chosen β, to 0.225 (bottom row), just over twice the original β. The prevalence in all hospitals increases with the increasing β, and the difference in prevalence between the hospitals persist.

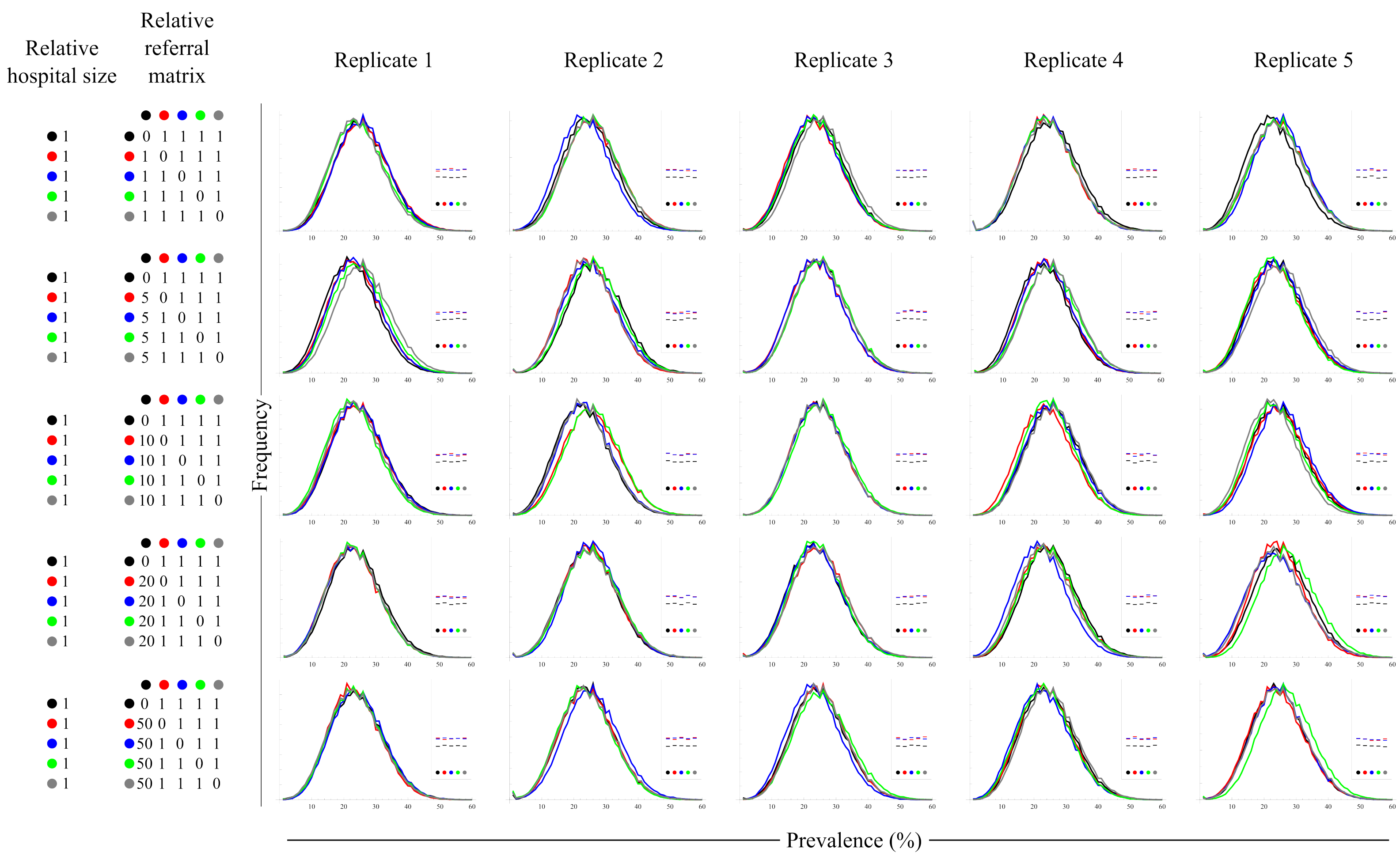

Figure S5: Reversion of the network direction by transposing the referral matrix. To check if the direction of the patient referral network can be reversed we created simulated datasets using the parameters from the networks with increasing directionality (See Directionality section). In this case, we used the transposed referral matrices as parameter. This does, however, results in a network without direction and equal prevalence in all hospitals, because the normalized referral probabilities are all equal.
